# Factor XII—A New Therapeutic Target? A Systematic Review

**DOI:** 10.3390/ijms27031331

**Published:** 2026-01-29

**Authors:** Katarzyna Krajewska, Joanna Pawlus, Katarzyna Ptaszynska, Anna Lisowska

**Affiliations:** 1 Department of Cardiology, Teaching Hospital in Bialystok, Medical University of Bialystok, 15-276 Bialystok, Poland; 2 Department of Haematology Diagnostics, Teaching Hospital in Bialystok, Medical University of Bialystok, 15-276 Bialystok, Poland

**Keywords:** factor FXII, factor in inflammation, FXII factor in heart failure, FXII in neuroinflammation, factor XII in coronary syndrome, factor XII in thrombosis

## Abstract

Factor XII is a molecule of unclear physiological function that has attracted increasing research interest across multiple medical disciplines. In recent years, a substantial body of evidence has emerged regarding the contribution of factor XII to the pathogenesis of inflammatory and prothrombotic conditions. FXII has been shown to play a protective role in FXII-driven coagulation during host defence against infections and to protect against multi-organ failure in animal models of sepsis. In acute respiratory distress syndrome (ARDS), FXII activity contributes to the release of pro-inflammatory mediators and is associated with severe clinical outcomes; it also induces fibroblast migration in idiopathic pulmonary fibrosis. FXII deficiency has been associated with reduced neutrophil adhesion and migration in sterile skin wounds and immune complex-induced vasculitis. In neurological conditions, FXII deficiency significantly reduced the number and severity of multiple sclerosis relapses and decreased the volume of post-traumatic brain oedema. In heart failure pathogenesis, FXII deficiency and pharmacological inhibition of FXII activity blocked activation of the renin–angiotensin–aldosterone system (RAAS) in dilated cardiomyopathy, increased median survival, and delayed heart failure onset in murine models. Importantly, FXII inhibition prevented arterial thrombosis without affecting haemostasis. This review summarises the latest findings on the contribution of FXII to inflammatory and prothrombotic states across multiple medical fields, including cardiology. Pharmacological inhibition of FXII has generated considerable interest as a potential future therapeutic strategy; however, to date, human studies remain limited.

## 1. Introduction

Factor XII (FXII), also known as the Hageman factor, was discovered by Oscar Ratnoff during routine blood testing of a patient named John Hageman, who was being prepared for surgical intervention and was found to have a markedly prolonged activated partial thromboplastin time (APTT) [1]. For many years, FXII was considered primarily in the context of the blood coagulation system, where it was recognised as a key component of the intrinsic coagulation pathway in plasma. Nevertheless, despite decades of research, the precise role of FXII in human physiology remains incompletely understood and continues to be a subject of intense scientific interest.

Human FXII is a glycoprotein produced predominantly in the liver; however, recent findings indicate the existence of an additional form secreted by leukocytes, which exhibits distinct functional properties [2]. FXII belongs to the family of serine proteases and circulates in plasma in two forms: the zymogen form (FXII) and its activated form, FXIIa. The zymogen form, long considered biologically inactive, is now known to exert multiple biological effects related to endothelial function and the activity of neutrophils, fibroblasts, and dendritic cells. Structurally, the FXII zymogen is a complex molecule composed of a light chain and a heavy chain. The heavy chain contains six distinct structural domains: an N-terminal fibronectin type II domain, an epidermal growth factor-like domain, a fibronectin type I domain, a second epidermal growth factor-like domain, a kringle domain, and a proline-rich region [3]. This complex molecular architecture, described in detail in the 1980s, prompted researchers to investigate potential non-coagulant and extra-proteolytic functions of FXII.

**Figure 1:**
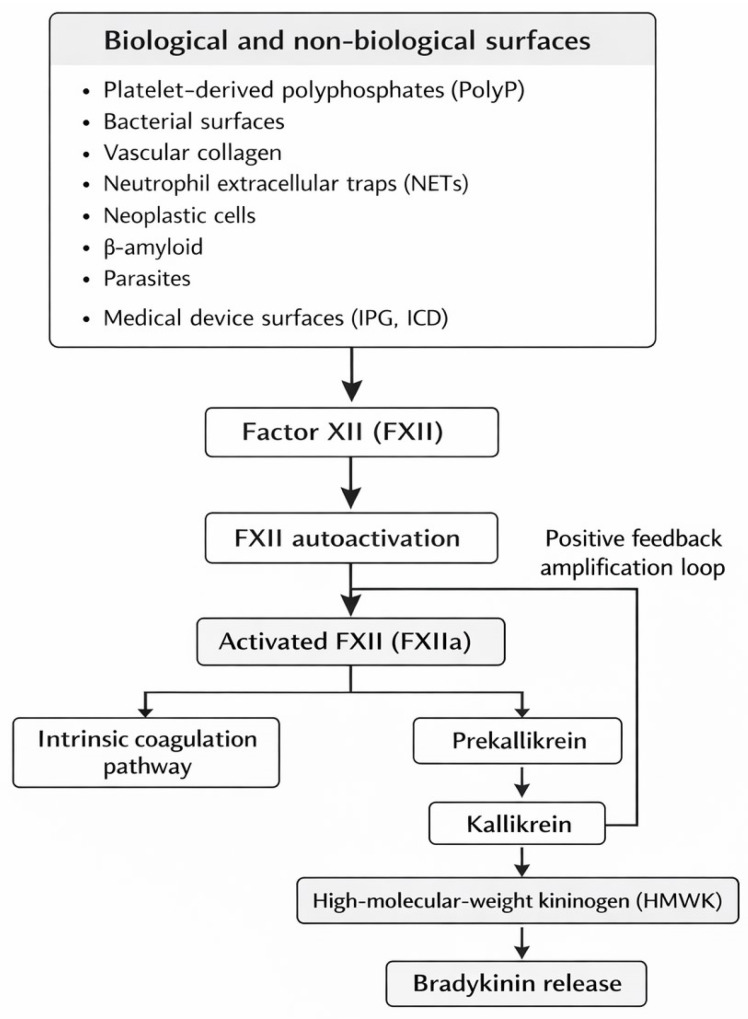
Contact system activation mediated by factor XII (FXII).

FXII activation, commonly referred to as classic contact activation, occurs in response to a wide range of biological and non-biological surfaces and substances. These include polyphosphates (PolyP) released from platelets, bacterial surfaces, vascular collagen, neutrophil extracellular traps (NETs), neoplastic cells, β-amyloid, parasites, and foreign surfaces of medical devices, such as the implantable pulse generator (IPG) and implantable cardioverter-defibrillator (ICD). FXII possesses the unique ability to undergo autoactivation, although the precise molecular mechanisms underlying this process remain unknown [2]. Upon activation, FXII initiates the intrinsic coagulation cascade; simultaneously, it catalyses the conversion of prekallikrein into active kallikrein, which, in turn, induces the release of bradykinin—a highly potent pro-inflammatory mediator—from high-molecular-weight kininogen (HMWK). Kallikrein further amplifies FXII activation through a positive feedback loop, resulting in a self-propagating activation process of the contact system (Figure 1) [2,4].

This review provides an overview of research on FXII conducted over the past decade, focusing on its role in inflammatory diseases across a broad range of medical disciplines, including neuroinflammatory disorders, pulmonology, allergology, cardiology, and other systemic conditions. The studies discussed herein indicate that inhibition of FXII may represent a promising therapeutic strategy in numerous diseases driven by chronic inflammatory processes. Particular interest has focused on the potential of FXII inhibition as a breakthrough approach in anticoagulant therapy, as congenital FXII deficiency does not impair haemostasis, making FXII an attractive target for thrombosis prevention without an increased risk of bleeding.

## 2. Materials and Methods

This review was conducted in accordance with the Preferred Reporting Items for Systematic Reviews and Meta-Analysis (PRISMA) guideline (Supplementary Materials) [5].

### 2.1. Eligibility Criteria

All original studies focusing on FXII, FXIIa, and bradykinin function and their outcomes in human and animal research were eligible for inclusion. These comprised observational, clinical, prospective, and cohort studies, as well as studies employing FXII-deficient animal models, pharmacological FXII blockade, assessment of FXII levels in human or animal plasma, and evaluation of FXII or bradykinin expression in neoplastic cell lines. Studies originated from a wide range of medical disciplines, including cardiology, neurology, surgery and cardiosurgery, pulmonology, nephrology, oncology, and infectious diseases.

### 2.2. Exclusion Criteria

All review papers and case reports were excluded from the review.

### 2.3. Information Source and Search Strategy

A systematic literature search was performed using the PubMed and Science Direct databases, covering publications from the past 10 years (June 2015 to June 2025) and open-access articles. The following Medical Subject Headings (MeSH) and keywords were used to identify relevant studies: “factor XII”, “inflammation”, “heart failure”, “coronary syndrome”, “thrombosis”, “neuroinflammation”, and “cancer”. These terms were combined using the Boolean operators AND/OR according to the following scheme. We first used “factor XII AND inflammation OR heart failure OR coronary syndrome OR thrombosis OR neuroinflammation OR cancer”, and separately used “factor XII AND inflammation, factor XII AND heart failure, factor XII AND coronary syndrome, factor XII AND thrombosis, factor XII AND neuroinflammation, factor XII AND cancer”. In addition, the reference lists of retrieved articles were manually screened to identify further relevant studies. Titles and abstracts were independently screened for relevance by two researchers (K.K. and J.P.). Additional records identified through manual searches of ResearchGate and the Google search engine were duplicates and, therefore, excluded. The study selection process is illustrated in Figure 1.

### 2.4. Data Items and Extraction

Data from studies that met the inclusion criteria were independently extracted by two reviewers (K.K. and J.P.). All eligible papers were collected in PDF format and screened in full. The following data were extracted: author name, country of origin, year of publication, study design, study population or experimental model, study aims, methods, and main findings. Extracted data were compiled into a table (Table 1) created using Microsoft Excel. Based on Table 1, studies were categorised according to the medical field.

### 2.5. Risk of Bias and Individual Studies

The risk of bias (ROB) was assessed in accordance with the Risk Of Bias In Non-Randomised Studies of Interventions (ROBINS-I) tool [6]. The following domains were evaluated: bias due to confounding; bias in the selection of participants; bias in the classification of interventions; bias due to deviations from intended interventions; bias due to missing outcome data; bias in outcome measurement; and bias in the selection of reported results. Each domain was rated as “Low”, “Moderate”, “Serious”, or “Critical” according to ROBINS-I criteria. Studies were classified as having a low overall risk of bias if all domains were rated as low risk, and as having some concerns if at least one domain was judged to present a concern. Assessments were conducted independently by two reviewers (K.K. and J.P.). In cases of disagreement, a third reviewer (A.L.) adjudicated. Study selection was supported using the Rayyan AI-assisted screening tool.

### 2.6. Literature and Sources

**Figure 2:**
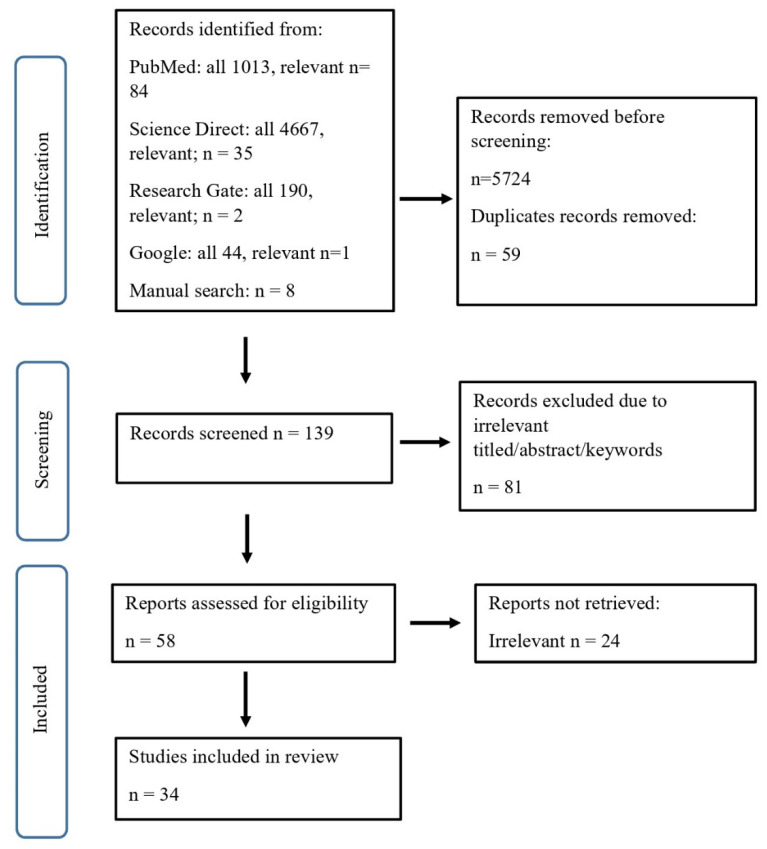
Flow diagram for the studies evaluated and included in the systematic review.

A total of 139 records were identified through database searches and manual screening. Overall, 81 records were excluded based on title, keywords, or abstract. Consequently, 58 articles were retrieved and assessed for eligibility, of which 24 were excluded for irrelevant data. Ultimately, 34 original studies were included in the final analysis (Figure 2).

### 2.7. Basic Characteristics of Included Trials

The general characteristics of the included studies are presented in Table 1. Our review included thirty-four studies that met the inclusion criteria. Those studies were mainly conducted on animal models, and several of them were conducted on humans or human plasma. The included studies originated from the following countries: 9 from the USA, 8 from Germany, 1 from Armenia, 1 from Australia, 1 from Brazil, 1 from France, 1 from Italy, 2 from the Netherlands, 1 from Spain, 2 from Sweden, 1 from Taiwan, and 6 from China. Eleven of them concerned neuroinflammatory and neurodegenerative diseases, nine concerned cardiac conditions, including thrombosis, mainly atrial fibrillation, one concerned sterile inflammation, two concerned chronic lung diseases, one concerned diabetic kidney disease, three focused on infectious diseases, one was related to hereditary angioedema, and six focused on cancer.

**Table 1:** General characteristics of included trials.

Author, Country, Study Design	Study Object/Population	Aim and Analysed Parameters	Main Results
Inflammatory response			
1. Evi X. Stavrou et al. 2018, USA [7]	Mice: FXII-deficient (F12−/−) and wild-type	The role of FXII in inflammatory response in models of sterile skin wounds and peritonitis.	FXII deficiency is associated with decreased neutrophil migration.
2. Pengfei Xu et al. 2024, China [8]	Mice	The effect of FXII inhibition with single-domain antibody (nanobody, Nb) on thrombus formation in (ECMO) and inflammation in vasculitis.	Nb-Fc treatment inhibited arterial thrombosis without affecting haemostasis and immune-induced local vasculitis.
3. A’lvaro Teijeira et al. 2020, Spain [9]	Murine cell cultures	The role of NETosis on tumour cell elimination by CD8^+^ T cells and natural killer cells (NK).	NETs protect tumour cells from cytotoxicity and promote tumour progression. NETosis inhibition had beneficial influence on effectiveness of tumour cell elimination.
4. Ahmed Elwakiel et al., 2024, Germany [10]	FXII-deficient (F12−/−) and wild-type mice	Pathogenic role of FXII in DKD.	FXII deficiency protected against hyperglycaemia-induced kidney injury.
5. Robert Silasi et al., 2021, USA [11]	Baboons	Analysis of the anticoagulant FXII-neutralising antibody in the mechanistic role of FXII in a baboon model of Staphylococcus aureus sepsis.	Inhibition of FXII prevented fever, terminal hypotension, respiratory distress, and multi-organ failure.
6. Katrin F. Nickel et al., 2025, Germany [12]	FXII-deficient (F12−/−) and wild-type mice	The role of FXII-driven coagulation pathway in host defence in bacterial infections on models of pneumonia induced with Streptococcus pneumoniae and Staphylococcus aureus skin infection.	A protective role for FXII-driven coagulation in host defence.
Chronic lung conditions			
7. Hess R. et al., 2017, Germany [13]	54 ARDS patients and 43 controls	Assessment of activation of the contact system, pro-coagulant activity, and inflammatory response in ARDS by measuring XIIa, kallikrein, bradykinin, IL-8, IL-1β, IL-6, CXCL5 and TNF-α in BALF.	Accumulation of FXII in ARDS lungs contributed to the release of pro-inflammatory mediators and was associated with clinical outcome.
8. Jade Jaffar, 2022, Australia [14]	Patients with IPF n = 27; healthy controls n = 35; 22 (62%) male, 8 (23%) female	Comparison of plasma levels of FXII and IL-6 and fibroblast migration in lung tissue in patients with IPF vs. control group.	FXII-induced IL-6 production and migration of fibroblasts in lung tissue.
Cancerogenesis			
9. Ying Zhou et al., 2019, China [15]	130 cervical cancer (CC) cases in mice	Measurement of bradykinin level in serum samples and BDKRB2 expression in tissue samples from patients with CC and CIN.	BK was overexpressed in patients with CC compared to patients with CIN and the control group.
10. Wei Wang et al., 2019, China [16]	Human cervical cancer cell lines	B2R expression detection in cervical cell lines.	BK promoted proliferation, migration, and invasion of CC cells.
11.Ding-Ping Sun et al., 2020, Taiwan [17]	Human malignant U87MG glioblastoma cell line; murine malignant glioblastoma cells	Evaluation of bradykinin role in migration and invasion of glioblastoma cells and possible mechanisms.	Bradykinin induced migration and invasion of human glioblastoma cells.
12. Micheli M Pillat et al., 2016, Brazil [18]	GBM cell lines	Evaluation of the role of kinin receptors for interactions between GBM cells and mesenchymal stem cells.	The role of BK in conveying information flow between these cells is important for tumour progression and invasion.
13. Yijie Chen et al., 2016, China [19]	Human HCC cell lines	Expression of BDKRB2, TRPM7, and MMP2 in human HCC cell lines.	BK promoted migration and invasion of HCC cells through TRPM7 and MMP2.
14. Guojun Wang et al., 2017, China [20]	Murine gastric cancer cell lines	BK in cell proliferation, migration, invasion, and tumour growth of gastric cancer.	BK promoted cell proliferation, migration, invasion, and the in vivo tumour growth of GC cells.
15. Katrin F. Nickel et al., 2015, Germany [21]	Mice	The effect of FXII inhibition on prostate cancer-mediated pro-coagulant activity.	Targeting polyphosphate or factor XII conferred resistance to prostate cancer-driven thrombosis, without increasing bleeding.
Neuroinflammatory response			
16. Kerstin Göbel et al., 2016, Germany [22]	FXII-deficient (F12−/−) mice CD87-deficient (Cd87−/−) mice	Impact of FXII deficiency on MS course.	FXII inhibition significantly reduced the number and severity of MS relapses.
17. Sarah Hopp et al., 2016, Germany [23]	Cortical cryogenic lesion model in mice	The impact of genetic deficiency of FXII and pharmacological inhibition of FXIIa with trauma-induced microvascular thrombus formation.	Genetic deficiency of FXII and pharmacological inhibition of FXIIa in mice minimises trauma-induced microvascular thrombus formation and improves outcome.
18. Sarah Hopp et al., 2017, Germany [4]	Cortical cryogenic lesion model in mice	The impact of genetic deficiency of FXII and pharmacological FXIIa inhibition on brain lesion size.	Genetic deficiency of FXII and pharmacological FXIIa inhibition diminish brain injury-induced lesion size.
19. Nicole Ziliotto et al., 2018, Italy [24]	Human plasma from: 74 MS patients and 49 healthy subjects	Assessment of FXII activity and FXII protein levels in MS patients.	MS patients present increased FXII protein levels and reduced function within the intrinsic coagulation pathway.
20. Kristina Johansson, 2017, Sweden Prospective Cohort Study [25]	1852 randomly selected participants; 165 MI, 108 with IS ischemic stroke, and 30 HS Female, n 938 (50.6%) Age, years 50 (38–62)	Investigation of the correlation between FXII and cardiovascular risk factors in the general population.	The significant association between FXII levels and haemorrhagic stroke.
21. Kristina Johansson et al., 2021, Sweden Prospective Case-Referent Study [26]	70 patients with first-ever ICH; 137 matched referents.	FXII plasma-level measurements due to association between factor XII and risk of ICH.	No association between factor XII and risk of ICH.
22. Daria Zamolodchikov et al., 2019, USA [27]	Human neuronal cell lines	FXII mRNA expression in the human brain.	A short isoform of FXII mRNA is expressed in the brain.
23. Daria Zamolodchikov et al., 2016, USA [28]	Human plasma; FXII-deficient (F12−/−) mice and wild-type mice plasma	The potential of Aβ to contribute to a prothrombotic state.	Aβ promotes coagulation via the FXII-driven contact pathway.
24. Zu-Lin Chen et al., 2017, USA [29]	AD mice and wild-type mice	The role of the contact system in AD pathogenesis.	Depletion of FXII in AD mice inhibited HK cleavage in plasma and reduced neuroinflammation and neurodegeneration in the brain.
25. Eugénie Garnier, 2021, France [30]	Mice	Assessment of neuroprotective roles of FXII and FXIIa in a model of neuronal injury induced by stereotaxic intracerebral NMDA injection.	FXII protects cultured neurons from apoptosis through a growth factor-like effect.
Cardiology/cardiosurgery			
26. Inna P. Gladysheva et al., 2024, USA [31]	DCM mice with FXII-deficiency; the DCM control group	Plasma renin activity in DCM mice vs. control group.	FXII deficiency and inhibition of FXII activity block activation of renin and RAAS.
27. Linghe Wu et al., 2023, Netherlands [32]	Atrial tissue obtained from autopsied COVID-19 16 patients with AF and 10 control patients	Analysis of inflammation, cardiomyocyte injury, and microvascular thrombogenicity in the atria of patients with COVID-19.	Increased presence of TF and FXII in the microvascular endothelium, dispersed cardiomyocyte injury, and presence of microvascular thrombosis in AF connected with COVID-19.
28. Lusine Hazarapetyan et al., 2023, Armenia [33]	283 patients with non-valvular AF; 183 patients after electrical cardioversion	The relationship between inflammation markers and coagulation activity in AF patients.	Patients with AF present a relationship between elevated levels of inflammatory markers and coagulation activity, which contributes to structural atrial remodelling.
28. Magdolna Nagy et al., 2024, Netherlands [34]	417 patients with PAF	The association of the activation of single coagulation factors with the progression of AF.	PAF FXIIa was inversely related to AF progression.
30. Jiachen Li et al., 2018, China Prospective observational study [35]	88 patients undergoing emergent surgery for AAD	Changes in intrinsic coagulation pathway during operation on AAD.	FXII might be an independent risk factor for reoperation for coagulopathy.
31. Frauke May et al., 2016, Germany [36]	Mice and rabbits	Assessment of the antithrombotic efficacy of the FXIIa inhibitor, rHA-Infestin-4, in several thrombosis models.	rHA-Infestin-4 prevented occlusion in the arterio-venous shunt model in mice and rabbits, where thrombosis was induced via a foreign surface.
32. Yasin Kokoye et al., 2016, USA [37]	FXII-deficient (F12−/−) and HK (Kng1−/−) mice	Estimation of the importance of FXII and PK to thrombosis.	Inhibitors of FXIIa have more potent antithrombotic effects than inhibitors of α-kallikrein.
33. Jun Wan et al. 2024, USA [38]	Plasma obtained from FXII-deficient (F12−/−) and wild-type mice	Evaluation of the pro-coagulant activity of prekallikrein in a mouse whole-blood thrombin-generation assay.	PKa contributes significantly to contact pathway-initiated thrombin generation.
Hereditary angiodema			
34. Timothy J Craig et al., 2023, USA [39]	80 patients with type I or type II HAE	The role of garadacimab as prophylaxis for HAE.	Garadacimab administration significantly reduced hereditary angioedema attacks.

Abbreviations: AAD—acute aortic dissection; AD—Alzheimer’s disease; AF—atrial fibrillation; ARDS—acute respiratory distress syndrome; Aβ—β-amyloid; B2R—bradykinin receptor 2; BALF—bronchoalveolar lavage fluid; BDKRB2—bradykinin receptor B2; BK—bradykinin; CC—cervical cancer; CIN—cervical intraepithelial neoplasia; CXCL5—C-X-C motif chemokine ligand 5; DCM—dilated cardiomyopathy; DKD—diabetic kidney disease; ECMO—extracorporeal membrane oxygenation; FXII—factor XII; GBM—glioblastoma; GC—gastric cancer; HAE—hereditary angioedema; HCC—hepatocellular carcinoma; HS—haemorrhagic stroke; ICH—intracerebral haemorrhage; IL-6—interleukin 6; IPF—idiopathic pulmonary fibrosis; IS—ischaemic stroke; MI—myocardial infarction; MMP2—matrix metalloproteinase-2; MS—multiple sclerosis; NETs—neutrophil extracellular traps; NMDA—N-methyl-D-aspartic acid; PAF—paroxysmal atrial fibrillation; RAAS—renin–angiotensin–aldosterone system; TNF-α—tumour necrosis factor α; TRPM7—transient receptor potential cation channel M7.

## 3. Results

### 3.1. The Function of FXII in Inflammatory Response

In the 1980s, researchers demonstrated that activated factor XII (FXIIa), via its heavy chain, influences neutrophil aggregation and degranulation [40]. Activated neutrophils produce a pool of FXII that is functionally distinct from liver-derived FXII and contributes to neutrophil migration towards sites of inflammation [7,41]. Neutrophil activation and FXII release initiate both inflammatory and thrombotic pathways. The interaction between these processes promotes persistent inflammation, sustained FXIIa generation, and thrombosis [7,41]. The synergistic pro-inflammatory and prothrombotic properties of FXII are, therefore, considered a key pathogenic mechanism underlying the conditions discussed below.

**Figure 3:**
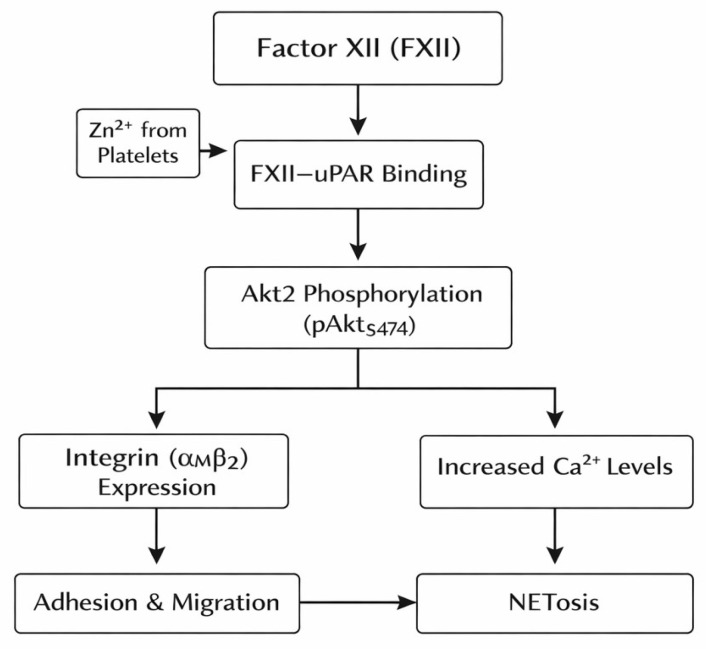
FXII–uPAR-dependent neutrophil activation and NETosis. The scheme illustrates FXII–uPAR-dependent neutrophil activation. Binding of coagulation factor XII (FXII) to the urokinase plasminogen activator receptor (uPAR), facilitated by zinc ions (Zn^2+^) released from platelets, leads to phosphorylation of Akt2 at serine 474 (pAktS474). Akt2 activation results in increased αMβ2 integrin expression and elevated intracellular calcium (Ca^2+^) levels in neutrophils, promoting adhesion, migration, and initiating the process of NETosis.

Neutrophil activation signalling is mediated by the interaction between FXII and the urokinase plasminogen activator receptor (uPAR). Binding of FXII to uPAR on endothelial cells is dependent on zinc ions released from platelets. This interaction induces phosphorylation of protein kinase Akt2 at serine 474 (pAktS474), which promotes expression of the αMβ2 integrin on the neutrophil surface. This integrin is essential for neutrophil adhesion, migration, and chemotaxis. In addition, FXII–uPAR signalling increases intracellular calcium levels, facilitating cell motility, and stimulates the process of NETosis, characterised by the extracellular release of neutrophil extracellular traps (NETs) composed of DNA, histones, and granule proteins aimed at the neutralisation of pathogens (Figure 3) [2,7,41].

#### 3.1.1. FXII in Chronic Wound Healing, Vasculitis, and Infections

Studies conducted on neutrophil migration used two sterile inflammatory models—cutaneous wound healing and chemically induced peritonitis—in FXII-deficient (F12−/−) and wild-type (WT) mice. In sterile wound models, FXII−/− mice exhibited impaired neutrophil migration and significantly faster wound healing. By day 7 of observation, 25% of FXII−/− mice had achieved complete wound closure (defined as <5% of the original wound area), whereas none of the WT mice had healed completely. Re-epithelialisation was significantly more advanced in FXII−/− mice compared with WT controls (70.3% ± 7% vs. 54.1% ± 4%, mean ± SEM; *p* = 0.04) [7]. Similar findings were observed in thioglycolate-induced peritonitis, where FXII−/− mice exhibited a reduced number of peritoneal exudative cells, with a significantly lower proportion of neutrophils compared with WT mice (9% vs. 39%) [7].

The role of FXII was further demonstrated in murine models of immune complex-mediated vasculitis and local ANCA-associated or anti-glomerular basement membrane (anti-GBM) vasculitis. Inhibition of FXII activity or genetic deletion of FXII reduced vascular inflammation and was associated with impaired neutrophil migration [8]. Overall, FXII-deficient mice exhibited diminished neutrophil adhesion, migration, and chemotaxis, as well as impaired angiogenesis.

FXII–uPAR interactions have also been implicated in diabetic kidney disease (DKD), where FXII promotes integrin β1-dependent signalling, leading to oxidative stress, DNA damage, and cellular senescence. FXII expression in renal tubular cells correlated with kidney dysfunction in murine models of DKD, whereas FXII deficiency conferred protection against hyperglycaemia-induced renal injury [10].

Pharmacological inhibition of FXII prevented fever, hypotension, respiratory distress, and multi-organ failure in baboons with sepsis induced by *Staphylococcus aureus* [11]. In contrast, another study demonstrated that FXII inhibition may facilitate the spread of infection by impairing local immune defence mechanisms. In murine models of lethal pneumonia caused by *Streptococcus pneumoniae* and skin infection induced by *Staphylococcus aureus*, FXII-deficient mice exhibited a more severe disease course, faster bacterial dissemination, and increased mortality. Similar effects were observed following pharmacological depletion of plasma prekallikrein [12]. Immunofluorescence analysis of abscess walls from FXII-deficient mice revealed a 50% reduction in fibrin content and a threefold increase in gap size compared with WT mice, resulting in defective abscess architecture and bacterial escape. In WT mice, bacteria remained confined within the abscess cavity [12]. These findings raise important concerns regarding the safety of FXII inhibition in the treatment of chronic wounds.

In the treatment of hereditary angioedema (HAE), a fully human monoclonal IgG4 antibody directed against the FXIIa/FXII inhibitor garadacimab has demonstrated promising efficacy [39].

#### 3.1.2. FXII in Acute and Chronic Lung Conditions

FXII is also produced by fibroblasts in the human lung. This process is induced by interaction between transforming growth factor β1 (TGF-β1) and phosphorylated c-Jun N-terminal kinase (p-JNK), resulting in increased FXII expression [42]. Hess et al. reported elevated FXII levels in bronchoalveolar lavage fluid from patients with acute respiratory distress syndrome (ARDS), which correlated with increased concentrations of pro-inflammatory interleukins (IL-8, IL-1β, and IL-6) and higher mortality rates [13].

In studies investigating the contribution of FXII to idiopathic pulmonary fibrosis (IPF), plasma FXII levels were not significantly elevated in patients with IPF compared with healthy controls (*p* > 0.05), in contrast to IL-6 levels. However, FXII expression was increased in IPF lung tissue, with a higher proportion of FXII/IL-6/α-smooth muscle actin-positive cells compared with healthy donors. FXIIa induced IL-6 expression in lung fibroblasts via protease-activated receptor-1 (PAR-1) and NF-κB signalling pathways, an effect that was completely mitigated by FXII-neutralising antibodies [14].

#### 3.1.3. FXII in Cancerogenesis

Cancer pathogenesis involves several mechanisms that constitute major challenges in oncological treatment. One of the key processes is fibroblast proliferation and tissue fibrosis mediated by transforming growth factor-β (TGF-β), which also induces the expression of factor XII (FXII). Another important component of the tumour microenvironment is the presence of neutrophils and the process of NETosis, which influences the elimination of cancer cells.

A study conducted in murine cell cultures demonstrated that FXII protected tumour cells from the cytotoxic effects of CD8^+^ T lymphocytes and natural killer (NK) cell-mediated lysis. The authors further showed that inhibition of NETosis enhanced the effectiveness of tumour cell elimination when combined with dual PD-1 and CTLA-4 immune checkpoint blockade [9]. Bradykinin, a product of FXII activation, plays a significant role in cancer development and functions as a growth-stimulating factor through interactions with bradykinin receptors B1R and B2R. This has been confirmed in studies on cervical and ovarian cancers, glioblastoma, and gastric and hepatocellular carcinoma cell lines [15,16,17,19,20]. The addition of bradykinin into cell cultures was shown to induce angiogenesis and increase vascular endothelial growth factor (VEGF) expression in cervical cancer cell lines [15]. Furthermore, activation of the STAT3 signalling pathway—known to promote proliferation and metastasis in multiple malignancies—was identified as a bradykinin-mediated mechanism contributing to cervical cancer progression [16]. Bradykinin has also been identified as a mediator of signalling between tumour cells and mesenchymal stem cells, facilitating tumour progression in malignant glioblastoma [18]. Pharmacological blockade of B1R or B2R was found to reduce tumour invasiveness in cervical cancer, malignant glioblastoma, and colorectal cancer [17,43]. Additionally, increased FXII expression has been observed in ovarian cancer compared with healthy ovarian epithelium, where it promoted peritoneal metastasis by inducing an M2-polarised phenotype in monocytes/macrophages [44]. In prostate cancer, continuous contact activation induced by circulating tumour cells contributes to increased pro-coagulant activity. Long-chain polyphosphates identified on the surface of prostate tumour cells have been shown to trigger coagulation in an FXII-dependent manner [21].

To summarise, based on available data, FXII inhibition presented promising results in sterile inflammation conditions, including sterile wounds, IPF, diabetic kidney disease, or neoplasms, but using this mechanism in infectious diseases is limited due to impaired fibrin trap formation and risk of bacteria spread.

### 3.2. FXII in Neuroinflammatory and Neurodegenerative Conditions

Autoimmune diseases of the central nervous system (CNS), such as multiple sclerosis (MS), are mediated by complex interactions between diverse cellular populations and immune components. Patients with MS have been shown to exhibit elevated plasma levels of factor XII (FXII) during disease relapse. In experimental autoimmune encephalomyelitis induced in FXI−/− mice, a reduced degree of inflammatory infiltration was observed, indicating a contribution of contact system components to neuroinflammatory processes [2]. Furthermore, FXII-mediated activation of dendritic cells has been demonstrated to modulate adaptive immune responses through urokinase plasminogen activator receptor (uPAR)-dependent cytokine production, thereby promoting neuroinflammation [2,22].

#### 3.2.1. FXII in Brain Injury and Post-Traumatic Brain Oedema

Trauma-induced inflammation and brain oedema are two key pathogenic mechanisms contributing to secondary brain injury and are mediated, in part, by the contact–kinin system. In patients following traumatic brain injury, a correlation between bradykinin levels and the extent of cerebral oedema has been observed [45]. Sarah Hopp and colleagues demonstrated that both genetic and pharmacological FXII deficiency exerted protective effects against thrombus formation, neurodegeneration, and cognitive impairment. Moreover, FXII inhibition resulted in improved therapeutic outcomes, including better motor function and a reduced volume of brain injury, without an increased risk of bleeding [23]. In a murine cryogenic lesion model, the same authors reported a significantly smaller lesion size in FXII-deficient mice and in mice treated with recombinant human albumin–Infestin-4 (rHA-Infestin-4) at 1 and 3 days post-injury compared with control animals [4]. Both genetic and pharmacological FXII inhibition were associated with reduced bradykinin release and attenuation of brain injury, blood–brain barrier (BBB) disruption, and oedema formation in mice.

In contrast, wild-type mice exhibited significantly higher levels of pro-inflammatory mediators, including interleukin-1β (IL-1β), tumour necrosis factor-α (TNF-α), CC-chemokine ligand 2 (CCL2), and increased expression of intercellular adhesion molecule-1 (ICAM-1), resulting in enhanced neutrophil and macrophage recruitment. These findings indicate that FXIIa-mediated bradykinin generation plays a crucial role in the pathogenesis of secondary brain injury through the amplification of inflammatory processes [4].

#### 3.2.2. FXII in Autoimmune Encephalomyelitis

Elevated FXII activity observed during relapse in patients with multiple sclerosis (MS) suggests that FXII may function as a neuromodulator. Increased FXII activity has been associated with a higher frequency of relapses and shorter intervals between relapse episodes, independently of immune-modulating therapies [22]. In experimental models, FXII-deficient mice immunised with myelin oligodendrocyte glycoprotein peptide 35–55 (MOG_35–55_) to induce experimental autoimmune encephalomyelitis (EAE) exhibited delayed disease onset and reduced clinical severity. These effects correlated with a decreased number of interleukin-17A-producing T cells. Impaired differentiation of Th17 cells was accompanied by reduced numbers of CD4^+^ and CD8^+^ T lymphocytes, as well as CD11b^+^ and CD11c^+^ cells, within the central nervous system of EAE mice. Reconstitution with human FXII fully restored susceptibility to EAE, confirming the direct role of FXII in disease pathogenesis [22]. Dendritic cells exposed to FXII produced increased levels of interleukin-6 (IL-6) and interleukin-23 (IL-23) in response to lipopolysaccharide (LPS) stimulation, compared with control cells, while the production of anti-inflammatory cytokines, including IL-10, IL-12, IL-27, and transforming growth factor-β (TGF-β), was significantly reduced. The Th17-mediated immune response was shown to depend on the interaction between the FXII and CD87 receptor, as demonstrated in experiments using CD87-deficient dendritic cells. Pharmacological inhibition of FXII with recombinant human albumin–Infestin-4 (rHA-Infestin-4) significantly reduced relapse frequency and disease severity, even when treatment was initiated after the first clinical manifestation of disease [22].

In line with the proposed neuromodulatory role of FXII, other investigators analysed the potential modulation of FXII activity using MS treatment strategies, including disease-modifying therapies (DMTs) and symptomatic treatments. This analysis did not reveal significant differences in plasma FXII activity between MS patients and healthy controls, and no differences were observed during relapse compared with remission. However, follow-up assessment after a four-month period demonstrated significantly reduced FXII activity and a decreased FXII activity-to-concentration ratio in MS patients, suggesting a contribution of activated FXII (FXIIa) to the MS phenotype [24].

#### 3.2.3. FXII in Haemorrhagic Stroke

Kristina Johansson et al. conducted a prospective analysis investigating the association between factor XII (FXII) levels and cardiovascular risk in a general population from Northern Sweden over the period from 1994 to 31 December 2011. Among 1852 randomly selected participants, 165 experienced myocardial infarctions (MIs), 108 had undergone ischaemic stroke (IS), and 30 experienced haemorrhagic stroke (HS) events [25].

The analysis demonstrated a significant association between FXII plasma concentration and the risk of haemorrhagic stroke only, with a hazard ratio (HR) of 1.42 per standard deviation (SD) increase in FXII levels (95% confidence interval [CI]: 0.99–2.05). In a multivariable model adjusted for age, sex, smoking status, body mass index (BMI), cholesterol levels, hypertension, and diabetes, elevated FXII concentrations remained significantly associated with an increased risk of haemorrhagic stroke (HR 1.51 per SD; 95% CI: 1.03–2.21) [25]. In contrast, a subsequent prospective case–referent study conducted by the same author in a larger cohort of participants did not identify a significant association between FXII levels and the risk of haemorrhagic stroke [46].

#### 3.2.4. FXII in Neurodegenerative Disease

Numerous studies have investigated the role of factor XII (FXII) in the development of neurodegenerative diseases, particularly Alzheimer’s disease (AD), the leading cause of dementia worldwide. A central pathological feature of AD is the accumulation of β-amyloid (Aβ) in the brain parenchyma; however, the precise mechanisms underlying its neurotoxicity remain incompletely understood. AD is also characterised by a prothrombotic state, which may contribute to neuronal dysfunction through disturbances in cerebral blood flow.

The influence of FXII on neuronal tissue remains largely unexplored, especially in light of recent findings demonstrating that a short isoform of coagulation factor XII mRNA is expressed by neurons in the human brain. This neuronal FXII isoform is thought to be involved in the regulation of apoptosis, which is a process that plays a critical role in the pathogenesis of many neurodegenerative disorders [27].

One line of research has examined the potential of Aβ to induce thrombotic processes through FXII-mediated fibrin generation in plasma derived from patients with AD. The addition of Aβ_42_ to platelet-rich or platelet-poor plasma resulted in dose-dependent thrombin generation, indicating that platelet-derived phospholipids rather than platelets themselves are required for this effect. Notably, this phenomenon was absent in plasma obtained from FXII-deficient mice. Aβ_42_ was shown to induce factor XI activation both in the presence and absence of prekallikrein, suggesting that Aβ_42_ acts as an FXII-independent activator of FXI [28,47]. The authors proposed a direct interaction between Aβ and fibrinogen/fibrin, leading to fibrin deposition within cerebral blood vessels. This process may contribute to microvascular occlusions, chronic neuroinflammation, and blood–brain barrier disruption through activation of the FXII-mediated coagulation cascade and increased bradykinin release [47].

In another study investigating the role of the contact system in AD pathogenesis, AD mouse models exhibited an age-dependent decline in plasma levels of high-molecular-weight kininogen (HK) and FXII, in contrast to wild-type mice, in which these levels remained stable. At six months of age, AD mice showed significantly increased expression of astrocytic and microglial/macrophage activation markers, including glial fibrillary acidic protein (GFAP) and ionised calcium-binding adaptor molecule 1 (Iba-1). A temporal correlation was observed between activation of the plasma contact system and the onset of neuroinflammation. Following antisense oligonucleotide (ASO)-mediated knockdown of the FXII gene, AD mice exhibited reduced fibrin deposition in the brain, attenuation of neurodegenerative changes, and improved cognitive performance [29].

#### 3.2.5. FXII in the Regulation of Neuroapoptosis

To elucidate the role of factor XII (FXII) and its activated form (FXIIa) in neuronal tissue, a group of investigators examined their effects on apoptosis using both in vivo and in vitro experimental models. An in vivo brain injury model was established by stereotaxic intracerebral injection of N-methyl-D-aspartate (NMDA), while apoptotic neuronal death in vitro was induced in murine neuronal cultures by serum deprivation.

Administration of FXII/FXIIa 10 min prior to NMDA injection resulted in a significant reduction in injury severity. Specifically, the mean lesion volume measured by histological analysis and magnetic resonance imaging (MRI) was reduced by 45% and 37%, respectively, compared with control animals at 24 h post-injection. The anti-apoptotic effect of FXII/FXIIa was further confirmed by immunofluorescence analyses demonstrating reduced apoptotic signalling.

Mechanistically, the neuroprotective effect of FXIIa is thought to be mediated by its interaction with the epidermal growth factor receptor (EGFR). Given previous evidence that FXIIa can also activate the hepatocyte growth factor (HGF) and hepatocyte growth factor receptor (HGFR) signalling pathway, the authors investigated a potential trophic mechanism by exposing serum-deprived neuronal cultures to FXIIa in the presence of an HGF-neutralising antibody. Under these conditions, the anti-apoptotic effect was completely abolished, indicating a critical role for HGF-mediated signalling. Importantly, this neuroprotective effect appeared to be independent of both fibrinolytic activity and the classical contact activation pathways [30].

In summary, FXII inhibition reduced severity of MS, minimised trauma-induced lesions and oedema, and improved clinical outcome. Moreover, it reduced neuroinflammation and neurodegenerative changes in AD.

### 3.3. FXII in Cardiology

To date, the role of factor XII (FXII) in cardiovascular disease has been investigated predominantly in the context of atrial fibrillation (AF) pathogenesis. In contrast, relatively few studies have examined the involvement of FXII in heart failure or in the setting of cardiac surgery.

#### 3.3.1. FXII in Heart Failure

The progression of heart failure (HF) is primarily driven by activation of the renin–angiotensin–aldosterone system (RAAS), the blockade of which constitutes a cornerstone of contemporary HF therapy. However, the precise mechanisms underlying renin activation in vivo remain incompletely understood. Previous studies have demonstrated increased plasma renin activity in patients and murine models with dilated cardiomyopathy (DCM) in the absence of chronic renal impairment [48,49]. Notably, elevated renin activity was found to correlate with increased plasma levels of factor XII (FXII).

In FXII-deficient DCM (DCM/FXII−/−) mice, administration of exogenous activated FXII (FXIIa) induced plasma renin activity that was approximately two-thirds higher than that observed in control animals. Conversely, a reduction in plasma FXII activity was associated with prolonged median survival and delayed onset of HF in mice. Furthermore, FXII deficiency in DCM mice was linked to improved cardiac contractility and a reduction in left ventricular dilatation, as reflected by increased ejection fraction, stroke volume, and cardiac output [31].

#### 3.3.2. FXII in Atrial Fibrillation (AF)

Post-mortem examinations conducted to analyse inflammation, cardiomyocyte injury, and biomarkers of microvascular thrombosis in atrial fibrillation (AF) associated with COVID-19 revealed significantly increased infiltration of lymphocytes, macrophages, and neutrophils within the atrial myocardium and epicardial adipose tissue compared with control subjects. Moreover, increased FXII activity and enhanced expression of tissue factor (TF) were observed in the microvascular endothelium of affected patients [32].

Patients with AF have also been shown to exhibit significantly elevated levels of pro-inflammatory markers, including high-sensitivity C-reactive protein (hs-CRP), interleukin-6 (IL-6), tumour necrosis factor-α (TNF-α), and increased levels of prothrombotic factors, including FXII (231.4 ± 65.4 vs. 125.4 ± 46.4; *p* = 0.033) [33]. In contrast, another study reported significantly lower plasma levels of FXIIa complexes in patients with progressive paroxysmal AF compared to individuals without AF. The authors were unable to definitively interpret these findings, but suggested that FXII may either exhibit heightened reactivity or exert protective effects in the context of AF pathophysiology [34].

#### 3.3.3. FXII in Cardiosurgery

In a single-centre study, investigators analysed changes in the intrinsic coagulation pathway during surgical treatment of acute aortic dissection (AAD) in 88 patients, of whom 24 required reoperations due to coagulopathy. Multivariable analysis demonstrated that perioperative FXII levels, as well as the administered doses of fibrinogen, red blood cells (RBCs), and fresh frozen plasma (FFP), were significantly higher in patients who required reoperation.

Based on these findings, the authors suggest that elevated FXII levels may represent an independent risk factor for reoperation due to coagulopathy, with an odds ratio (OR) of 1.342 (95% confidence interval [CI]: 1.058–1.570; *p* = 0.012) [35].

#### 3.3.4. FXII and Contact System in Thrombosis

In a murine model of thrombosis induced by ferric chloride (FeCl_3_), FXII-deficient mice were markedly resistant to thrombus formation and carotid artery occlusion, even at higher concentrations of FeCl_3_. Specifically, no thrombosis was observed in FXII-deficient mice following exposure to a 5% FeCl_3_ solution, and only partial occlusion occurred at a 10% concentration, whereas wild-type mice developed carotid artery occlusion at concentrations as low as 3.5%. Mice deficient in high-molecular-weight kininogen (HMWK) also demonstrated partial resistance to thrombosis at a 5% FeCl_3_ concentration [37]. In human plasma treated with prothrombotic stimuli such as DNA, collagen, or silica, antibodies targeting FXIIa were more effective at preventing thrombus formation than antibodies directed against α-kallikrein [37].

Furthermore, a single-domain antibody fused to the Fc region of human immunoglobulin (Nb–Fc), which inhibits FXII activation, demonstrated a protective effect against thrombosis in mice without increasing the risk of bleeding complications [8]. In a murine extracorporeal membrane oxygenation (ECMO) model, FXII inhibition significantly reduced the formation of clots on the membrane surface as well as the occurrence of microvascular thrombi. Consistently, in vitro experiments using human blood showed that Nb–Fc prevented collagen- and neutrophil activation-induced fibrin deposition [8].

In conclusion, FXII inhibition blocked RAAS activation. FXIIa inhibitors had antithrombotic effects. Increased intrinsic system activity was related to AF progression.

#### 3.3.5. Inhibition of FXII as an Anticoagulant Therapy

Currently, several lines of research are focused on factor XII (FXII) as a potential therapeutic target for anticoagulation. The principal strategies under investigation include the following:

- FXIIa inhibitor rHA-Infestin-4—Recombinant human albumin–Infestin-4 (rHA-Infestin-4) is a selective FXIIa inhibitor. In preclinical studies conducted in mice and rabbits, this agent conferred protection against ischaemic stroke and ferric chloride-induced occlusive carotid artery thrombosis triggered by exposure to artificial surfaces, without increasing the risk of bleeding complications [27,36].
- Antisense oligonucleotide (ASO)—Antisense oligonucleotides targeting FXII reduce hepatic synthesis of proteins by promoting degradation of FXII-encoding mRNA. Experimental studies in mice demonstrated effective protection against both venous and arterial thrombosis without an associated bleeding risk. Additionally, ASO-mediated FXII suppression prevented catheter-associated thrombosis in rabbit models [50,51].

Comparative studies suggest that inhibition of factor XI (FXI) may provide superior antithrombotic efficacy compared with FXII inhibition in primate models of venous thromboembolism (VTE) and ischaemic stroke prevention [52,53]. Conversely, FXII inhibition appears to play a particularly important role in preventing thrombosis associated with artificial surfaces, which is a finding that aligns with the physiological function of FXII in contact activation pathways [53].

## 4. Discussion

The functions of factor XII (FXII) have been the subject of increasing scientific interest in recent years. This review focused on recent advances in understanding the contribution of FXII to inflammatory and prothrombotic processes implicated in a wide range of pathological conditions across multiple medical disciplines. The available evidence highlights FXII as a central mediator linking coagulation, inflammation, and immune responses. Pharmacological inhibition of FXII has emerged as a promising therapeutic strategy, attracting considerable attention as a potential approach for the prevention and treatment of thrombo-inflammatory disorders. The unique position of FXII within the contact activation system suggests that its modulation may offer antithrombotic efficacy with a reduced risk of bleeding, supporting further clinical investigations.

Effective wound repair requires the coordinated interaction of multiple cell populations. Wound healing proceeds through three overlapping phases: the inflammatory phase, the proliferative phase, and the remodelling phase. This ultimately results in scar formation [54]. A critical component of this process is neutrophil activity, owing to antimicrobial functions mediated by phagocytosis and the release of pro-inflammatory cytokines. These mediators promote angiogenesis and stimulate the proliferation of keratinocytes and fibroblasts [55]. Upon activation, neutrophils release extracellular DNA structures known as neutrophil extracellular traps (NETs), which serve as a substrate for FXII activation and subsequent fibrin trap formation, thereby limiting the spread of infection. The duration of the inflammatory phase is crucial for appropriate wound healing, as its prolongation is associated with continued recruitment of inflammatory cells, excessive neutrophil activity, and the development of chronic non-healing wounds [7,41,56]. Signalling pathways mediated by the urokinase plasminogen activator receptor (uPAR) promote endothelial cell proliferation and angiogenesis in vivo, exerting effects comparable to those of vascular endothelial growth factor (VEGF) [2]. Interestingly, studies using non-infected wound models have demonstrated that FXII deficiency may be beneficial, potentially through impaired neutrophil migration. Chronic wounds represent a significant clinical challenge, particularly in patients with venous or arterial peripheral vascular disease, poorly controlled diabetes mellitus, and other comorbidities. In clinical practice, many of these wounds are complicated by difficult-to-treat infections, frequently caused by multidrug-resistant organisms. In light of evidence suggesting a protective role of FXII in local infections via fibrin trap formation, important questions arise regarding the safety of pharmacological FXII inhibition in the treatment of chronic wounds. The findings of the studies discussed appear partly contradictory, and no comparable investigations have yet been conducted in humans. Consequently, it remains uncertain whether pharmacological blockade of FXII is safe with respect to the risk of pathogen dissemination and systemic infection. There is a clear need for further studies examining the impact of FXII inhibition on infection risk in both non-infected wounds undergoing treatment and wounds that are already infected. Based on fluorescence imaging studies, FXII has been proposed to function as a chemotactic factor. Current data indicate that FXIIa accounts for approximately 50% of plasma bradykinin generation. Bradykinin is a key inflammatory mediator responsible for regulation of vascular tone, as well as the production of nitric oxide, prostacyclin, and tissue plasminogen activator (tPA). Nevertheless, reduced neutrophil recruitment to skin windows was not observed in mice genetically deficient in bradykinin receptors or in animals treated with the bradykinin antagonist R715. Finally, uPAR-mediated cytotoxic effects on tubular cells—which can be attenuated by pharmacological inhibition of FXII—may also have implications for the prevention of diabetic kidney disease, providing an additional rationale for future investigation in this area [10].

Experimental blockade of activated factor XII (FXIIa) has been shown to reduce post-traumatic brain oedema mediated by bradykinin release. The resultant reduction in injury volume was associated with improved motor performance and enhanced cognitive function. These findings suggest that FXII may exert a protective role within the central nervous system (CNS), at least in part, through anti-apoptotic mechanisms. Studies investigating the role of FXII in neuroapoptosis indicate that both FXII and FXIIa indirectly influence neuronal survival via epidermal growth factor receptor (EGFR)- and hepatocyte growth factor receptor (HGFR)-dependent signalling pathways, which modulate gene expression involved in the regulation of apoptosis. These observations raise important considerations regarding the safety of FXII inhibition, despite the highly promising therapeutic outcomes observed in models of post-traumatic brain injury and experimental autoimmune encephalomyelitis [4,22,23].

Pharmacological blockade of factor XII (FXII), as a key mediator of bradykinin release, may represent a potential therapeutic strategy in the treatment of various malignancies. However, this approach requires careful investigation with respect to its possible infectious consequences.

Particularly intriguing are the potential benefits of FXII inhibition in systemic chronic diseases, including cardiovascular and pulmonary conditions such as idiopathic pulmonary fibrosis, chronic obstructive pulmonary disease (COPD), and other connective tissue disorders manifesting with pulmonary fibrosis. IPF is an irreversible and progressive disease that leads to chronic respiratory failure and is associated with a poor prognosis and high mortality rate. Owing to the close functional and anatomical relationship between the lungs and the heart, chronic pulmonary diseases frequently result in cardiovascular complications, most notably pulmonary hypertension (PH). PH associated with lung disease represents the second most prevalent form of pulmonary hypertension, following PH related to left heart failure. The most common pulmonary causes of PH include COPD and pulmonary fibrosis associated with systemic sclerosis (SSc). Increased pulmonary vascular resistance leads to pressure overload of the right ventricle, ultimately resulting in symptomatic right heart failure. In this context, inhibition of FXII or downstream signalling pathways such as nuclear factor kappa B (NF-κB) may attenuate interleukin-6 (IL-6) expression, suppress fibroblast proliferation, and thereby slow disease progression and reduce mortality. Effective pharmacological blockade of FXII could, therefore, represent a novel therapeutic strategy and a potential alternative or adjunct to existing antifibrotic agents, such as nintedanib, currently used in the treatment of IPF.

At present, relatively limited data are available regarding the role of factor XII (FXII) in cardiovascular diseases. Its involvement has been primarily investigated in the context of inflammatory and prothrombotic mechanisms underlying atherosclerosis and atrial fibrillation, in which FXII has been shown to contribute to the inflammatory background of these conditions. In contrast, evidence concerning the role of FXII in the pathogenesis of heart failure (HF) remains scarce. Heart failure has been described by some investigators as an epidemic of the twenty-first century, with an estimated prevalence of 1–2% of the population in highly developed countries. In 2024, a group of American researchers published findings that provided novel insights into the pathomechanisms of renin activation mediated by FXII. This discovery suggests a potential new therapeutic target for modulation of the renin–angiotensin–aldosterone system (RAAS), complementary to established approaches such as angiotensin-converting enzyme inhibition (ACEI). The involvement of FXII in HF pathogenesis, therefore, represents an innovative and promising area for further investigation. The studies discussed herein support the biological activity of FXII as a potential therapeutic target not only in preventing thrombosis and modulating inflammatory responses, but also in the inhibition of pathogenic mechanisms driving HF progression.

Another important aspect of FXII in cardiology relates to anticoagulant therapy. Currently available anticoagulants used in clinical practice predominantly target factor II (thrombin) and factor X, which are key components of the common coagulation pathway. Since the discovery that congenital FXII deficiency does not result in clinically significant haemostatic defects, pharmacological inhibition of FXII has emerged as an attractive strategy for thrombosis prevention without an increased risk of bleeding [7]. However, evidence supporting the efficacy of FXII inhibitors is currently limited to preclinical animal models. In one such study, an antibody targeting FXIIa was more effective at preventing thrombosis than an antibody directed against α-kallikrein. Notably, inhibitors of factor XI (FXI) are being investigated more extensively than FXII inhibitors, which may, in part, reflect technical challenges associated with obtaining crystal structures of FXIIa. Experimental data suggest that FXI inhibition may provide superior antithrombotic efficacy compared with FXII inhibition in primate models of venous thromboembolism (VTE) and ischaemic stroke. Conversely, FXII inhibition may play a particularly important role in preventing thrombosis associated with artificial surfaces, consistent with the physiological role of FXII in contact activation. Furthermore, elevated FXII levels have been proposed as an independent risk factor for reoperation due to coagulopathy in the setting of cardiac surgery, underscoring its potential clinical relevance in cardiovascular medicine.

Available data provide a wide range of promising information. Future investigations are necessary to fully understand the role of this mystery molecule in vivo, possibly in human models, and to explore security of its pharmacological blockade.

## 5. Conclusions

Elevated plasma activity of factor XII (FXII) has been documented across a broad range of pathological conditions. Available evidence suggests that targeting FXII may exert disease-modifying effects by simultaneously attenuating pro-inflammatory and prothrombotic pathways in disorders such as Alzheimer’s disease, post-traumatic brain injury, and sterile inflammatory conditions, including chronic wound healing. FXII inhibition may also offer therapeutic benefits in limiting the progression of idiopathic pulmonary fibrosis (IPF) and ovarian cancer, as well as in preventing thrombosis associated with prostate cancer [14,21]. Furthermore, FXII appears to play a significant role in the course of heart failure through modulation of the renin–angiotensin–aldosterone system (RAAS), and its inhibition may be particularly effective at preventing thrombosis related to artificial surfaces. Collectively, these findings highlight the FXII axis as a promising target for future therapeutic strategies and a compelling subject for further investigation. Nevertheless, additional research is required to fully elucidate the physiological and pathophysiological roles of FXII in vivo, particularly in human disease models [7,57].

## Data Availability

No new data were created or analyzed in this study. Data sharing is not applicable to this article.

## Supplementary Materials

The following supporting information can be downloaded at https://www.mdpi.com/article/10.3390/ijms27031331/s1. Reference [58] is cited in the Supplementary Materials.
